# Activation of Metabotropic Glutamate Receptors Regulates Ribosomes of Cochlear Nucleus Neurons

**DOI:** 10.1371/journal.pone.0111243

**Published:** 2014-10-21

**Authors:** Kathryn L. Carzoli, Richard L. Hyson

**Affiliations:** Program in Neuroscience, Department of Psychology, Florida State University, Tallahassee, FL, United States of America; University of Washington, Institute for Stem Cells and Regenerative Medicine, United States of America

## Abstract

The brain stem auditory system of the chick is an advantageous model for examining changes that occur as a result of deafness. Elimination of acoustic input through cochlear ablation results in the eventual death of approximately 30% of neurons in the chick cochlear nucleus, nucleus magnocellularis (NM). One early change following deafness is an alteration in NM ribosomes, evidenced both by a decrease in protein synthesis and reduction in antigenicity for Y10B, a monoclonal antibody that recognizes a ribosomal epitope. Previous studies have shown that mGluR activation is necessary to maintain Y10B antigenicity and NM viability. What is still unclear, however, is whether or not mGluR activation is sufficient to prevent deafness-induced changes in these neurons, or if other activity-dependent factors are also necessary. The current study investigated the ability of mGluR activation to regulate cochlear nucleus ribosomes in the absence of auditory nerve input. *In vitro* methods were employed to periodically pressure eject glutamate or mGluR agonists over neurons on one side of a slice preparation leaving the opposite side of the same slice untreated. Immunohistochemistry was then performed using Y10B in order to assess ribosomal changes. Application of glutamate and both group I and II selective mGluR agonists effectively rescued ribosomal antigenicity on the treated side of the slice in comparison to ribosomes on the untreated side. These findings suggest that administration of mGluR agonists is sufficient to reduce the early interruption of normal ribosomal integrity that is typically seen following loss of auditory nerve activity.

## Introduction

Sensory experience plays an essential role in establishing and refining neural organization during development. In fact, abnormal input or experiential modifications due to injury can produce severe changes in neuronal morphology including alterations in dendritic length, cell size, and can even influence the fate of a cell [1]–[4]. Second-order auditory neurons in the chick brain stem have proven useful in investigating the importance of sensory experience during development [5]. Neurons in the cochlear nucleus, nucleus magnocellularis (NM) receive their sole excitatory input from the cochlea via the ipsilateral eighth nerve [6], [7]. Thus, unilateral cochlear ablation results in a complete loss of excitatory afferent input to the ipsilateral NM but leaves the contralateral side unaffected, allowing for within-subject comparisons of NM neurons on the intact versus the deafened side of the brain. Elimination of auditory nerve activity during a sensitive period of development leads to the death of approximately 20–30% of NM neurons on the deafened side of the brain [8]. The remaining neurons survive, albeit at a lower metabolic rate and with a reduction in soma size [8]–[12].

The factors that determine which cells die and which cells survive still remain unclear; however, the chain of events that occur within NM as a consequence of deafening are well documented. Within 1 to 3 hrs following cochlear ablation, NM neurons show a threefold increase in intracellular calcium ([Ca^2+^]_i_) [13], and a decrease in RNA and protein synthesis [12], [14], [15]. At 6–12 hrs, deafened NM neurons appear to segregate into two populations: one population suffers a complete cessation of protein synthesis and eventually goes on to die, while the other population continues to synthesize proteins, albeit at a reduced level, and goes on to survive [12]. The cessation of protein synthesis appears to be due to the dissociation of polyribosomes in NM neurons following cochlear ablation [16]. One way to visualize the rapid activity-dependent changes that occur in ribosomes following deafening is by using Y10B, a monoclonal antibody that recognizes a ribosomal epitope [14], [15], [17], [18]. Changes in Y10B immunoreactivity match the changes observed in overall protein synthesis [17], and these changes correspond with changes observed in ribosomes at the EM level [15], [16]. Consequently, Y10B immunoreactivity provides an efficient and meaningful assay for examining changes in NM ribosomes, one of the earliest cellular changes to occur following deafferentation.

Studies directed at identifying the trans-synaptic signals necessary for preventing the early changes that occur in NM neurons following cochlea removal have made use of an *in vitro* slice preparation of the chick auditory brain stem. In this condition, NM activity is eliminated bilaterally since both cochleae are destroyed. In order to mimic the case of unilateral deafening, auditory nerve fibers are electrically stimulated on one side of the slice while fibers on the opposite side of the same slice remain unstimulated. Within 1 hr, NM neurons on the stimulated side of the slice show greater protein synthesis [19] and Y10B labeling [18], [20], [21] than the neurons on the unstimulated side. Through the use of the *in vitro* slice preparation, it has been shown that glutamate's action on ionotropic glutamate receptors (iGluRs) is responsible for the electrical activity in postsynaptic NM neurons [20], [22], and glutamate's action on metabotropic glutamate receptors (mGluRs) is responsible for providing trophic support to NM neurons [21], [23], [24]. Previous *in vivo* studies have also shown that mGluR activation is necessary for maintaining ribosomal antigenicity of Y10B and for the ultimate survival of NM neurons [25]. For example, application of selective group I or group II mGluR antagonists results in NM neuronal cell death even with an intact cochlea.

Although it is clear that mGluR activation is necessary to preserve NM neurons in a healthy state, it is not known if mGluR activation is sufficient to maintain these neurons or if other activity-dependent factors are also required. The present experiments evaluate whether or not activating mGluRs on deafferented NM neurons is sufficient for maintaining ribosomes in a healthy state during the period immediately following deafferentation. If adding mGluR activation to the deprived NM is sufficient to regulate NM neuronal ribosomes, then application of mGluR agonists should preserve ribosomal Y10B antigenicity, which is normally lost in the absence of auditory experience.

## Materials and Methods

### Subjects

All subjects were 1 to 4 day old chicks of either sex, hatched from eggs obtained from a local supplier and reared at Florida State University. The procedures used in these experiments were approved by the Animal Care and Use Committee at Florida State University and conform to the guidelines set forth by the National Institutes of Health. All efforts were made to minimize the number of animals used and their suffering.

### Slice preparation

Subjects were anesthetized with isoflurane and decapitated. A 4 mm segment of the caudal skull containing the brain stem was removed with a razor blade, quickly submerged in room temperature artificial cerebral spinal fluid (ACSF), and was oxygenated using a 95% O_2_, and 5% CO_2_ gas mixture. ACSF consisted of (in mM) 130 NaCl, 3 KCl, 2 CaCl_2_, 2 MgCl_2_, 26 NaHCO_3_1.25 NaH_2_PO_4_ and 10 dextrose. The brain stem was rapidly dissected from the skull segment and mounted onto a custom-built stage for slicing using cyanoacrylate glue with additional support provided by a 30% gelatin compound. Using a vibrating blade microtome, a 300 µm, bilaterally symmetrical, coronal slice containing NM was obtained, transferred to a submersion-type recording chamber, and perfused (2–3 ml/min by gravity) with oxygenated ACSF. The slice was anchored in the recording chamber using nylon-strung metal “harp”.

### Drug administration

Once the brain slice was placed in the chamber, a glass micropipette was lowered into the bath and positioned over the slice toward the lateral side of the IVth ventricle. Drugs were ejected onto one side of the brain slice using periodic pressure pulses (10 psi, 10–50 msec duration) through a picospritzer (General Valve Corporation, Fairfield, N.J.). The slice was oriented such that the laminar flow of ACSF through the chamber carried the drug only to NM on the outflow side of the chamber. Different groups of slices (n = 4 per group) were unilaterally treated with either vehicle, glutamate (1 mM), the general mGluR agonist, ± trans 1-amino-1,3-cyclopentanedicarboxylic acid (ACPD) (400 µM), a selective group I mGluR agonist, 3,5-dihydroxyphenylglycine (DHPG) (200 µM), or a selective group II mGluR agonist, LY354740 (200 nM) for 1 hr. Drug concentrations in the pipette were selected based on their EC_50_ and predicting an approximately10-fold reduction in concentration by the time the drug flowed to NM. A dose-response curve for glutamate was constructed using concentrations of L-glutamic acid ranging from 0.01 to 10 mM in the ejection pipette (n = 3–4 slices per group). The vehicle was dextrose-free ACSF.

### Confirmation of unilateral drug application

**Figure 1:**
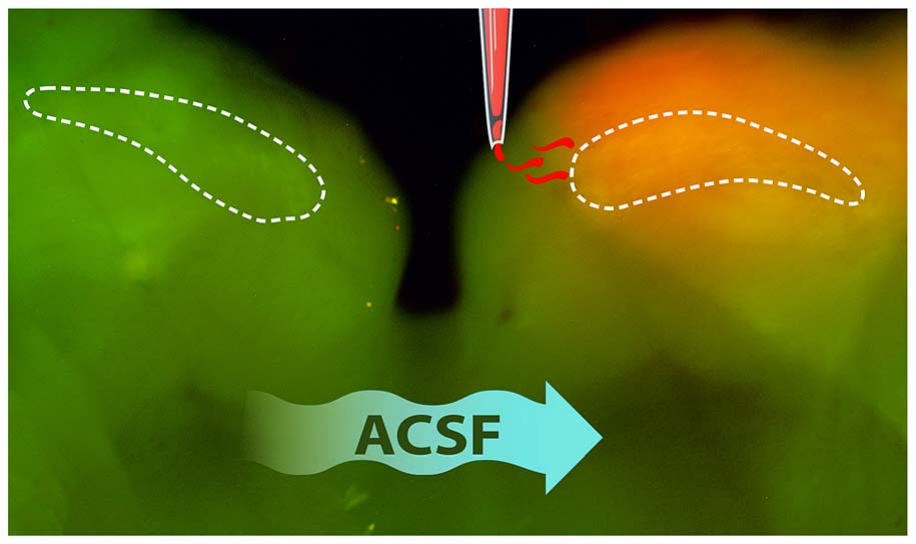
Fluorescence image of a brain stem slice containing nucleus magnocellularis (NM) schematically showing the pipette placement at the lateral side of the IV^th^ ventricle and demonstrating the distribution and spread of focally administered drugs. Arrows indicate the direction of the laminar flow of fluid in the chamber. White outlines indicate location of NM. This image of Fluoro-Ruby application confirms that agents that are applied using this method will reach NM unilaterally with minimal back-flow affecting the opposite side of the same section.

Preliminary tests using fast green were performed to monitor the distribution of solution released by pressure application in order to ensure drug was being administered to only one side of the slice and that minimal backflow was affecting the opposite side of the same slice. Additional confirmation was obtained by ejecting a 1% solution of a fluorescent dextran, tetramethylrhodamine (fluoro-ruby) 10,000 MW (Invitrogen), from the micropipette. Distribution of dye clearly showed that ejected substances primarily, if not exclusively, reached only one NM (see Figure 1). Dye was used in slices to document the application procedure and was not included for drug-treated slices, so as not to interfere with immunoreactivity.

### Tissue preparation

At the end of the 1-hr period of unilateral drug application, a notch was cut into the ventral portion of the side of the slice that received drug. The section was then post-fixed in 4% paraformaldehyde for 1 hr and cryoprotected in a 30% sucrose/phosphate-buffered saline (PBS) solution overnight.

Since all comparisons were performed between opposite sides of the same tissue section, it was necessary to preserve symmetry between sides. This was accomplished by assuring that the slice was mounted flat for sectioning. A mound of tissue freezing medium was built up onto a cryostat chuck to approximately ½ inch in height. The chuck was then loaded into the cryostat and the top of the mound was sectioned off to obtain a flat surface. The 300 µm tissue slice was then placed onto a silanized microscope slide and excess fluid was removed with a kimwipe. The cryostat chuck was then placed on dry ice and a small amount of tissue freezing medium was deposited onto the flattened surface. The slide holding the brain slice was placed tissue-side-down into the fresh medium and a pellet of dry ice was placed on top of the slide to ensure rapid freezing. The microscope slide was then detached using a razor blade, leaving the brain slice embedded in the freezing medium. The mounted brain slice was allowed to equilibrate in the cryostat at −20°C for at least 30 min prior to sectioning. Using a Leica CM 1850 cryostat (Leica Microsystems Inc., Bannockburn, IL, USA), 20 µm sections were then collected from the mounted slice and were free-floated in a vial containing ice-cold PBS.

### Immunohistochemistry

Following sectioning, endogenous peroxidase activity was quenched by placing sections in 0.03% H_2_O_2_ in methanol for 20 min. After quenching, sections were rinsed 3×10 min in PBS, and were then preincubated in a 4% normal horse serum solution for 10 min. Sections were then incubated on a rotator overnight at room temperature in 1∶500 Y10B, a mouse monoclonal antibody that recognizes a ribosomal epitope. The Y10B antiserum was raised from a clone originally developed by J. Steitz and subsequently supplied to our laboratory by E. Rubel. This antibody is an established marker of changes in ribosomes [26], and has been used extensively as an indicator of the early changes resulting from deafferentation (e.g., [15], [17], [18], [25], [27]). Changes in antigenicity appear to match changes in overall protein synthesis and a deafferentation-induced reduction in Y10B labeling has been confirmed at the EM level [15]. Control sections processed without primary antibody show no labeling. The following day, sections were washed 3×10 min in PBS then incubated for 1 hr in 1∶200 biotinylated horse anti-mouse secondary antibody. Sections were washed 3×10 min in PBS and then incubated in avidin-biotin-peroxidase complex (Vectastain ABC Kit, Vector Laboratories, Burlingame, CA, USA) for 1 hr. Following 2×10 min washes in PBS and a 10 min wash in 0.1 M phosphate buffer (PB), sections were reacted with diaminobenzidine tetrahydrochloride and 0.03% H_2_O_2_ for 15 min. Sections were mounted onto slides following 2 10-min washes in 0.1 M PB, and allowed to dry overnight. The next day, slides were cleared though graded ethanols and xylenes and then coverslipped using DPX mounting medium (Sigma-Aldrich, St. Louis, MO, USA).

### Densitometry

To objectively analyze the level of immunolabeling, densitometric measurements of NM neurons were obtained using a digital image analysis program (NIH ImageJ). The staining densities of NM neurons on the drug-treated versus the untreated side of the same tissue section were compared. This within-subjects method improves statistical power. Although immunohistochemistry cannot provide quantitative differences in the amount of protein changed, it has crucial advantages over other methods (e.g., Western analysis) by selectively evaluating changes in neurons and examining if changes occur in all neurons or only a subpopulation of cells.

All comparisons were between opposite sides of the same tissue section. This assures that variation in labeling intensity is attributable to the experimental treatment and not to variation in immunohistochemistry processing variables (e.g., incubation time in any of the reagents). For these analyses, the light levels and camera settings remained constant. Cells were visualized using a 40X objective and mean gray scale densities over approximately 40 NM neurons/side in a given tissue section were measured beginning with cells at the medial edge of NM and proceeding laterally. At least 3 sections from each subject were measured. The investigator analyzing the tissue remained blind to the identities of the drug-treated and the untreated sides of the section as well as to the drug group until after measurements were obtained. Differences in the density of labeling between sides of the slice that received ACSF or drug were compared using analysis of variance (ANOVA).

## Results

**Figure 2:**
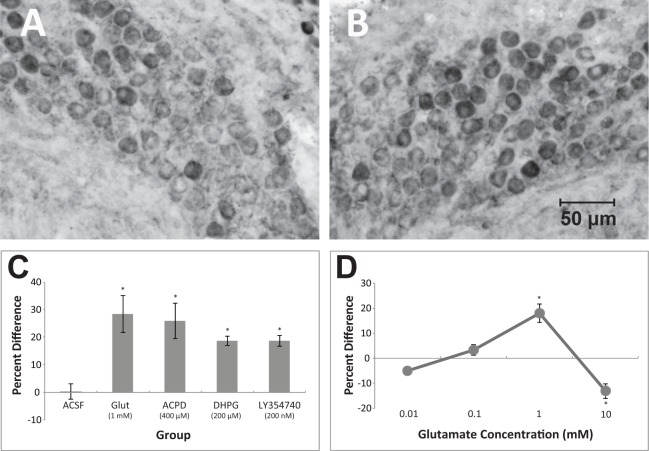
Photomicrograph comparing Y10B labeling between untreated (A) and mGluR agonist-treated (B) sides of the same brain slice. In this example, the group I selective mGluR agonist, DHPG, was applied to NM on one side of the slice for 1 hr. Tissue sections were immunolabeled with Y10B, a monoclonal antibody that recognizes a ribosomal epitope. There is darker Y10B immunolabeling of NM neurons on the drug-treated side of the brain section. (C) Percent differences in gray scale density for Y10B labeling between NM neurons on untreated versus drug-treated sides of the same brain slice ((mean treated density-mean untreated density)/mean treated density*100). Bars indicate standard error of the mean. Positive numbers indicate that the treated side had greater labeling for Y10B. Different groups of slices (n = 4 per group) received unilateral application of vehicle, L-glutamic acid, a general mGluR agonist, ACPD, an mGlu group I agonist, DHPG, or an mGlu-group II-selective agonist, LY354740. The vehicle-treated control slices showed no difference in Y10B labeling between sides while treatment with each agonist resulted in a darker Y10B labeling on the drug-treated side of the slice. (D) Dose-response curve plotting the mean percent difference in gray scale density for Y10B labeling following unilateral application of varying concentrations of L-glutamic acid. Positive numbers indicate that the treated side had darker Y10B immunolabeling. As concentration in the ejection pipette increased, the treated NM neurons first showed darker Y10B immunolabeling (at 1 mM) and then showed lighter immunolabeling (at 10 mM). The percent difference in labeling between opposite sides of the same slices was statistically lower at 10 mM than at any other concentration and the percent difference at 1 mM was statistically greater than at 0.01 mM but only marginally greater (p = 0.06) than the 0.1 mM group. These data suggest that the effect of glutamate transitions from neuroprotection at moderate concentrations, to neurotoxicity at high concentrations.

Vehicle treatment to NM on one side of the slice for 1 hr had no apparent effect on Y10B immunoreactivity. Application of glutamate (1 mM) resulted in darker Y10B immunolabeling as did the general mGluR agonist, ACPD 400 µM. Relatively low concentrations of the group I mGluR agonist, DHPG (200 µM), and the potent group II mGluR agonist, LY354740 (200 nM), also resulted in darker Y10B labeling on the drug-treated side of the slice in comparison to the side of the slice that remained unexposed to drug. An example of this effect can be seen in Figure 2A, B.

Visual impressions were confirmed through objective analyses of labeling density. The labeling densities of individual NM neurons on the drug-treated versus the untreated side of the same tissue slice were compared. A two-way mixed ANOVA was performed on the gray scale measurements using side of the section as the within-subject variable and drug as the between-subjects variable. This analysis revealed no reliable effect of drug treatment (F (4, 15) <1), an overall effect of side (F (1, 15)  = 52.55, *p*<0.001) and, importantly, a reliable drug treatment X side interaction (F (4, 15)  = 5.16, *p*<0.01). Post hoc (Newman-Keuls) pair-wise comparisons revealed that all agonist-treated groups showed a reliable difference between sides (p<0.05) whereas control-treated slices demonstrated no reliable difference between sides (see Figure 2C).

The protective effect of glutamate is somewhat surprising given its known excitotoxic effects in other systems (for review see [28]). Consequently, a dose response curve was generated over a broad range of concentrations (0.01 to 10 mM) in the application pipette. A relatively low concentration of L-glutamic acid (0.01 mM) produced no difference in Y10B labeling between the glutamate-treated and untreated side of the same slice. Treatment with 0.1 mM glutamate showed only a hint of an effect, but treatment with 1 mM glutamate replicated the first experiment and clearly yielded darker ribosomal labeling in NM neurons on the treated side of the slice compared to neurons on to the untreated side of the same slice. While the percent difference between sides following 1 mM glutamate treatment was slightly lower that that obtained in the first experiment, this difference between experiments was not statistically significant (t-test, p = .22). A high concentration (10 mM) had the opposite effect, in that the glutamate had detrimental effects on Y10B labeling on the glutamate-treated side of the slice when compared to the untreated side of the same slice.

Visual impressions were confirmed through objective analyses of staining density. The staining densities of individual NM neurons on the glutamate-treated versus the untreated side of the same tissue slice were compared. A two-way mixed ANOVA was performed on the gray scale density measurements using side of brain as the within-subjects variable and glutamate concentration as the between-subjects variable. Neither main effect of side (F (1,10)  = 0.34, p<1)) nor effect of concentration (F (3,10)  = 3.34, p = 0.063)) was statistically significant. Importantly, there was a statistically significant Side X Concentration interaction (F (3,10)  = 20.08, p<0.0001). To further compare the effect of glutamate concentration between groups, density measurements were normalized across brains by transforming gray scale scores to percent difference ((treated-untreated)/treated *100). A one-way ANOVA on the percent difference scores revealed a reliable dose effect (F (3,13)  = 13.3, p<0.001). Post hoc (Newman-Keuls) pair-wise comparisons (p<0.05) revealed that the percent difference in labeling for the 1 mM group was significantly higher than the 0.01 mM group but was only marginally different from the 0.1 mM group (p = 0.06). The 10 mM group showed a statistically reliable percent difference in labeling between sides, albeit in the opposite direction of all other groups (see Figure 2D).

## Discussion

It is widely accepted that early experience is essential to normal brain development. Atypical sensory input during development can have dramatic and potentially damaging effects on the central nervous system that can persist into maturity. A number of classic experiments have demonstrated this by restricting the amount of experience an animal receives through a single sensory modality. Cell death is observed following sensory deprivation in the visual [29], somatosensory [30], olfactory [31]–[34], vestibular [35], and auditory systems of both chicks and mammals [1], [2], [8], [36], [37]. Despite this common finding across developing sensory systems, relatively little is known about the trophic mechanisms that govern such activity-dependent cell survival.

The brain stem auditory system of the chick has proven to be a useful model system for examining the transneuronal signals necessary for activity-dependent cell survival. This is because deafening is relatively easy to produce in the chick. Moreover, the large cochlear nucleus cells form a relatively uniform population of neurons that undergo rapid and robust changes following deafferentation, leading to the ultimate death of a subpopulation of these cells. One early alteration that occurs following deafness in NM neurons is a change in ribosomal activity [12]. This change in function corresponds with a reduction in antigenicity for Y10B, a monoclonal antibody that recognizes a ribosomal epitope [14], [15], [17], [19], [26]. A number of studies have made use of the Y10B assay to examine the transneuronal signals necessary for the activity-dependent regulation of NM neurons. Earlier studies, have shown that electrical activity of the postsynaptic NM neuron is not sufficient to maintain NM neuronal ribosomes [18], [19], suggesting activity-dependent release of some important trophic factor from the active auditory nerve terminal. However, blockade of iGluR receptors, which are responsible for driving the electrical activity of NM neurons [22], does not prevent the activity-dependent regulation of ribosomal antigenicity [20]. This implies that neither iGluR activation nor postsynaptic electrical activity is necessary to keep NM neurons in a healthy state. On the other hand, blockade of mGluRs does prevent this activity-dependent regulation [21], [23]. The importance of mGluR activation has also been confirmed *in vivo* where it has been shown that blockade of mGluRs reduces ribosomal antigenicity and produces NM neuronal death even with an intact cochlea [25]. The current set of experiments demonstrate that providing mGluR activation to NM neurons is sufficient, at least in the early stages that follow deafferentation, to maintain healthy ribosomes. Application of glutamate, a general mGluR agonist (ACPD), a selective group I mGluR agonist (DHPG), or a selective group II mGluR agonist (LY354740), effectively preserved ribosomal antigenicity in NM neurons on the treated side of the slice in comparison to those on the untreated side. Together with previous studies of this system, it appears that mGluR activation is both necessary and, in part, sufficient to provide trophic support to NM neurons, which undergo degenerative changes following the loss of afferent activation that results from deafness.

One overall limitation of the studies discussed thus far is that an *in vitro* slice preparation isolates an individual brain slice from the rest of the body. This allows for the possibility that alternative sources of trophic support have also been eliminated in the slice preparation. Consequently, it is not known if mGluR activation is truly required for maintaining neuronal integrity in the intact system or if mGluR activation is only required when other forms of trophic support are no longer present. If such factors exist, however, they are released independent of auditory nerve activity, since there is no spontaneous activity in either the slice preparation or *in vivo* following cochlea ablation. Since the activity-dependent regulation of ribosomes is observed *in vitro*, any such co-factors are not circulating in the cerebrospinal fluid *in vivo*. The prospective co-factor is also unlikely to be glutamate acting at iGluRs since the present studies used selective agonists for mGluRs and previous studies have shown no effect of total iGluR blockade [20]. Consequently, if a co-factor is required, it is present in deafferented NM *in vitro* and could be any number of substances provided by glial cells or by synaptic release from neuron terminals that is independent of auditory nerve action potentials.

### MGluRs and neuroprotection

There are various mechanisms by which the different mGluRs could provide for neuroprotection, and these mechanisms can be generally categorized as having either direct or indirect effects on the neuron. One of the direct effects by which mGluRs could be protecting NM neurons is through the control of Ca^2+^ homeostasis. Manipulations of auditory nerve input to NM neurons and *in vitro* pharmacological treatments produce changes in [Ca^2+^]_i_ that correlate with the Y10B assay used in the present set of experiments. For example, in the absence of activity there is a rapid rise in [Ca^2+^]_i_, which can be reversed by electrical stimulation of the auditory nerve or mGluR activation [13], [24]. Additionally, blockade of mGluRs results in a dramatic rise in [Ca^2+^]_i_, even if afferent activity is provided [24]. It is possible that a change in Ca^2+^ homeostasis is a triggering event that results in the disruption of ribosomes observed in the present experiments.

There are unusually high demands placed on chick cochlear nucleus neurons with respect to Ca^2+^ homeostasis. This is because NM neurons see some of the highest rates of activity in the CNS, even in the absence of acoustic stimuli [38]. This requires NM to be equipped with a variety of compensatory mechanisms to help maintain the fine balance of intracellular Ca^2+^ levels. One such mechanism could involve mGluR activation by glutamate overspill that occurs when the usual uptake systems become overwhelmed [39], [40]. In fact, this has been shown to be the case in several brain regions containing glutamatergic neurons that fire at high frequency [41], [42].

Once activated, mGluRs are known to regulate Ca^2+^ through a number of signal transduction cascades. For example, mGluR activation of the adenylate cyclase cascade in NM can directly inhibit Ca^2+^ influx through voltage-operated calcium channels [43] via adenosine 3′,5′-cyclic monophosphate activation of protein kinase A, while activation of mGluRs that stimulate the phospholipase C cascade can indirectly regulate [Ca^2+^]_i_ through the generation of IP_3_ [44], which subsequently liberates Ca^2+^ from internal stores [45] and activates protein kinase C.

In the present studies, selective agonists for group I and group II mGluRs were able to reduce the loss of antigenicity for Y10B on the side of the slice that was treated with agonist compared to the untreated side of the same slice. Additionally, the combined effect of activating both mGluR groups through use of the non-selective mGluR agonist, ACPD, appeared to be more beneficial than the activation of specific groups, suggesting that activation of both I and II subgroups of mGluRs are important for the regulation of NM ribosomes. There is a chance, however, that the differences in effect seen between drugs could simply be due to variations in the effective potency of the concentrations used and the possibility of synergistic effects was not directly evaluated.

A possible indirect mechanism by which mGluR activation could lead to protection is by regulating inhibitory influences on NM neurons [46]. Activation of mGluRs has been shown to suppress GABA release to NM neurons, while blockade of mGluRs reportedly increases GABA release [47], [48]. GABA-ergic transmission in neurons of the avian auditory brainstem is unusual in that it is depolarizing but inhibitory [46], [49]. Membrane depolarizations activate voltage-gated Ca^2+^ channels and lead to Ca^2+^ influx, which could be detrimental to the cell. However, mGluR activity may effectively prevent GABA-evoked depolarizations, serving as yet another mechanism by which NM neurons buffer intracellular Ca^2+^.

Finally, mGluR activation could also be exerting a neuroprotective effect by working at neighboring glial cells. Previous research in the chick auditory brainstem has demonstrated that there is rapid growth of astrocytic processes in NM following cochlea removal [50]–[52]. The neuroprotective effect of mGluRs on glial cells has been documented in several systems. For example, stimulation of cortical glial cells via application of mGluR agonists has been shown to be highly neuroprotective in mixed cultures that have been exposed to toxic levels of NMDA [53]. It has also been suggested that substances released from glial cells in the hippocampus can influence local synapses [54]. If this is the case in NM neurons, then it is possible that mGluR activation could promote the release of some trophic substance from astrocytes.

### Glutamate concentration dependence and technical considerations

Although NM neurons are equipped with compensatory mechanisms that allow them to deal with higher levels of glutamate, the current experiments demonstrated that they are not immune to the toxic properties of glutamate. The ability of glutamate to regulate ribosomal antigenicity was contingent on concentration. Reliably darker labeling was observed when 1 mM glutamate was ejected into the media upstream of NM neurons, while a high concentration of glutamate (10 mM) had the opposite effect, yielding lighter labeling on the drug-treated side of the slice when compared to the control side of the same slice.

These findings are not unexpected since it is well established that glutamate has neurotoxic properties when released in large amounts or when incompletely recycled (for review see [28]). Studies looking at the role of glutamate transporters have shown that clearing glutamate from the synaptic cleft is an important regulatory control of synaptic strength and that this modulation could be traced to mGluRs located on glial cells [55]. It has also been shown that there is a proliferation of glial processes in NM following the cessation of auditory nerve activity [50]. Perhaps the important aspect of mGluR activation is on glial cells, which then modulate the uptake of potentially toxic levels of glutamate.

While the current experiment used a glutamate concentration of 1 mM in the ejection pipette, focal application through periodic pressure ejection allows for dilution of the drug. A disadvantage of this method of application is the uncertainty about the degree of drug dilution once expelled into the bath, which makes it impossible to know the precise drug concentration at the cell. Whole bath application would have allowed for more accurate dosimetry, but short pressure applications reduce receptor desensitization, and this method of agonist delivery more closely mimics the natural periodic activation by the endogenous transmitters. Similar to the present studies, bath superfusion of the mGluR agonist, ACPD has been shown to prevent the increase in [Ca^2+^]_i_ that occurs in the absence of auditory nerve stimulation, but unlike the present studies, bath superfusion of 1 mM glutamate has been shown to result in an increase in [Ca^2+^]_i_ [24]. This apparent discrepancy could either be due to the additional dilution of drug in our application procedure, bringing the 1 mM pipette solution to sub-toxic levels, or it could be due to some receptor desensitization in the studies that applied drugs in the perfusate, thereby preventing the receptors from having a protective influence and disrupting Ca^2+^ homeostasis.

## Conclusions

In summary, it is clear that activation of mGluRs protects NM neurons from early degenerative changes, both in the presence and absence of auditory nerve stimulation. Future research will have to determine the exact nature of this protective influence but there are likely multiple mediators. Activation of these receptors can modulate glutamate uptake at the auditory nerve-NM synapse, keeping glutamate at sub-toxic levels, or it could more directly protect the neuron by maintaining intracellular Ca^2+^ homeostasis, and perhaps act through a myriad of other modulatory functions that are regulated by 2nd messenger systems.
